# Clinical significance of tacrolimus intra-patient variability on kidney transplant outcomes according to pre-transplant immunological risk

**DOI:** 10.1038/s41598-021-91630-4

**Published:** 2021-06-09

**Authors:** Eun Jin Kim, Soo Jin Kim, Kyu Ha Huh, Beom Seok Kim, Myoung Soo Kim, Soon Il Kim, Yu Seun Kim, Juhan Lee

**Affiliations:** 1 Department of Surgery, Yonsei University College of Medicine, Seoul, Republic of Korea; 2 Department of Internal Medicine, Yonsei University College of Medicine, Seoul, Republic of Korea

**Keywords:** Kidney, Predictive markers, Risk factors

## Abstract

High intra-patient variability (IPV) of tacrolimus trough concentrations is increasingly recognized as a predictor of poor long-term outcomes in kidney transplant. However, there is a lack of information regarding the association between tacrolimus IPV and graft outcomes according to immunological risk. We analyzed tacrolimus IPV using the coefficient of variability from months 6–12 after transplantation in 1080 kidney transplant recipients. Patients were divided into two immunological risk groups based on pre-transplant panel reactive antibodies and donor-specific antibodies. High immunological risk was defined as panel reactive antibodies ≥ 20% or the presence of donor-specific antibodies. The effects of tacrolimus IPV on graft outcomes were significantly different between low and high immunological risk patients. A multivariable Cox regression model confirmed that high tacrolimus IPV was an independent risk factor for graft failure in the high risk group (HR, 2.90; 95% CI, 1.42–5.95, *P* = 0.004). In the high risk group, high tacrolimus IPV was also significantly associated with increased risk of antibody-mediated rejection (*P* = 0.006). In contrast, death-censored graft survival and antibody-mediated rejection in the low immunological risk group was not significantly different by tacrolimus IPV. High tacrolimus IPV significantly increases the risk of graft failure and antibody-mediated rejection in patients with high immunological risk.

## Introduction

Despite significant advances in short-term outcomes, long-term kidney transplantation (KT) outcomes remain suboptimal^1,2^. Beyond the first year post transplantation, approximately 3–5% of grafts are lost annually. Although the causes of late graft loss are multifactorial, alloimmune-mediated injury and adverse effects of immunosuppressive medications are major contributors^3–5^. As human leukocyte antigens (HLA) are the most important alloantigens in transplantation, transplant recipients have varying immunological risks according to donor-recipient HLA matching and previous sensitization. Therefore, tailored immunosuppressive treatment for a given patient with varying immunological risks remains a critical unmet need^6–8^. Unfortunately, current immunosuppressive treatment is based on center-specific protocols rather than the immunological risk profile of a given patient^9^.

The immunosuppressant tacrolimus is the cornerstone of the immunosuppression regimen in solid organ transplantation to prevent graft rejection and graft loss^10^. However, tacrolimus has a narrow therapeutic window with high inter- and intra-patient variability (IPV), requiring close monitoring of blood trough concentrations^11^. Patients with high tacrolimus IPV may be at risk of underexposure and alloimmune-mediated injury or overexposure and toxicity. A growing body of evidence suggests that high tacrolimus IPV is associated with poor graft outcomes^12,13^. Therefore, tacrolimus IPV is not only a useful tool to identify patients with a greater risk but also one of the most important modifiable risk factor for long-term graft outcomes^14^.

Tailored immunosuppressive strategy involves finding the lowest effective dose of immunosuppressive medication to control the alloimmune response while minimizing drug toxicity^15^. Immunological risk of individual patients is a key determinant for tailored immunosuppressive treatment^16^. In this context, the clinical significance of tacrolimus IPV should be assessed according to immunological risk. However, there is a lack of information regarding the clinical significance of tacrolimus IPV on graft outcomes according to immunological risk. In the present study, we evaluate the association between tacrolimus IPV and graft outcomes and rejection in low- and high immunological risk patients.

## Results

### Baseline characteristics

After implementing the inclusion/exclusion criteria, 1080 patients who underwent KT with tacrolimus-based immunosuppressive therapy were included in this study. Patients were divided into low- and high immunological risk groups according to peak panel reactive antibodies (PRA) and presence of donor-specific anti-HLA antibodies (DSA; high immunological risk was defined as PRA ≥ 20% or the presence of DSA). The baseline characteristics of patients are presented in Table 1. Compared to the low-risk group, immunologically high-risk patients were more likely to be older, female, and have longer dialysis vintage. The proportion of re-transplant cases and deceased donor KTs were significantly higher in the immunologically high-risk group than in the low-risk group. The median peak PRA of the high-risk group was 54% (IQR, 30.0–84.0). Of the high-risk group patients, 70 had pre-transplant DSA (22.4%). No significant differences in HLA mismatch, donor age, and tacrolimus formulation were observed between the two groups. The use of anti-thymocyte globulin for induction was significantly more common for patients in the high-risk group than for those in the low-risk group. The median follow-up duration was 82 months (IQR, 48.0–122.8).

**Table 1:** Baseline characteristics of patients.

Variables	Low immunological risk (N = 763)	High immunological risk (N = 317)	*P*
Female, *n* (%)	242 (31.7)	188 (59.3)	< 0.001
Age, years	45.1 ± 12.1	47.9 ± 11.6	< 0.001
Body mass index, kg/m^2^	22.9 ± 3.4	22.0 ± 3.2	< 0.001
**Mismatch HLA-A, B, DR**			0.661
1–2	170 (22.3)	78 (24.6)	
3–4	454 (59.5)	180 (56.8)	
5–6	139 (18.2)	59 (18.6)	
Peak %PRA, median (IQR)	0 (0–2.0)	54 (30.0–84.0)	< 0.001
Re-transplant, *n* (%)	43 (5.6)	58 (18.3)	< 0.001
Dialysis vintage, months	38.0 ± 52.3	63.0 ± 62.4	< 0.001
Deceased donor, *n* (%)	184 (24.1)	149 (47.0)	< 0.001
Female donor, *n* (%)	401 (52.6)	136 (42.9)	0.004
Donor age, years	43.2 ± 12.0	44.6 ± 13.3	0.120
**Induction agent,** ***n*** ** (%)**			
No	41 (5.4)	1 (0.3)	< 0.001
Basiliximab	660 (86.5)	240 (75.7)	
Anti-thymocyte globulin	62 (8.1)	76 (24.0)	
**Mean TAC IPV, CV%**	23.0 ± 10.4	22.5 ± 9.8	0.503
High TAC IPV, *n* (%)	146 (19.1)	62 (19.6)	0.872
Mean TAC concentration, ng/mL	6.2 ± 2.0	6.3 ± 1.9	0.464
Dose of TAC, mg/day	4.1 ± 2.2	4.0 ± 2.0	0.493
TAC concentration to dose ratio	2.1 ± 1.9	2.1 ± 1.4	0.865
**TAC formulation**			0.100
Twice daily TAC	644 (85.4%)	273 (89.2%)	
Once daily TAC	110 (14.6%)	33 (10.8%)	
**Co-medication**			
HMG CoA reductase inhibitor	386 (50.6%)	171 (53.9%)	0.315
Proton pump inhibitor	46 (6.0%)	30 (9.5%)	0.044
Diuretics	72 (9.4%)	53 (16.7%)	0.001
Anticoagulants or antiplatelet drugs	90 (11.8%)	54 (17.0%)	0.021

Values are expressed as n (%), mean ± SD or median (IQR) depending on the data type.

*HLA* Human leukocyte antigen, *PRA* Panel reactive antibodies, *TAC* Tacrolimus, *IPV* Intrapatient variability, *CV* Coefficient of variability.

### Tacrolimus trough level and IPV

A total of 9059 tacrolimus trough concentrations were analyzed. The overall median number of trough concentration measurements per patient between 6 and 12 months after KT was 8.0 (IQR, 7.0–9.0). The mean tacrolimus trough level was 6.2 ± 2.0 ng/mL for the entire cohort, 6.2 ± 2.0 ng/mL for low immunological risk patients, and 6.3 ± 1.9 ng/mL for high immunological risk patients (*P* = 0.464). The median tacrolimus IPV was 21.0% (IQR, 15.9–27.6) for the entire cohort, 21.0% (IQR, 16.0–27.7) for the low-risk cohort, and 21.0% (IQR, 15.6–27.5) for the high-risk group. The proportion of patients with high tacrolimus IPV [coefficient of variation (CV) > 30%] was not significantly different between the two groups (19.1% vs. 19.6%). There was no significant difference in tacrolimus concentration to dose ratio between the two groups.

To investigate potential risk factors associated with high tacrolimus IPV, we performed multivariable logistic regression analysis (Table 2). Recipient age, sex, BMI, tacrolimus trough level, serum albumin, and renal function were not associated with high tacrolimus IPV. Low hematocrit levels at 12 months post-transplantation, proton pump inhibitor, and high tacrolimus concentration to dose ratio were significantly associated with high tacrolimus IPV.

**Table 2:** Risk factors associated with high tacrolimus IPV.

Variables	Univariate		Multivariate	
	OR (95% CI)	*P*	OR (95% CI)	*P*
Elderly recipient (Age ≥ 60 years)	1.012 (0.645–1.588)	0.958		
Female	0.956 (0.701–1.303)	0.775		
Body weight, kg	0.992 (0.979–1.005)	0.208		
Body mass index, kg/m^2^	0.981 (0.937–1.027)	0.407		
High immunological risk group	1.044 (0.749–1.456)	0.799		
TAC concentration to dose ratio	1.129 (1.043–1.222)	0.003	1.143 (1.049–1.246)	0.002
Once daily tacrolimus formulation	0.855 (0.535, 1.366)	0.513		
**Laboratory findings at 12 months**				
Hematocrit, %	0.934 (0.908–0.960)	< 0.001	0.942 (0.914–0.971)	< 0.001
Albumin, mg/dL	1.036 (0.993–1.080)	0.105	1.034 (0.990–1.080)	0.133
eGFR < 60 mL/min/1.73m^2^	1.294 (0.951–1.759)	0.101	1.111 (0.790–1.562)	0.545
Proton pump inhibitor	2.349 (1.424, 3.874)	0.001	1.936 (1.114, 3.364)	0.019
Diuretics	1.378 (0.886, 2.144)	0.154	0.966 (0.585, 1.595)	0.892
Antiplatelet or anti-coagulant	0.963 (0.615, 1.508)	0.868		
HMG CoA reductase inhibitor	0.821 (0.606, 1.111)	0.202		

*TAC* Tacrolimus, *eGFR* Estimated glomerular filtration rate.

### Tacrolimus IPV and graft outcomes

**Figure 1:**
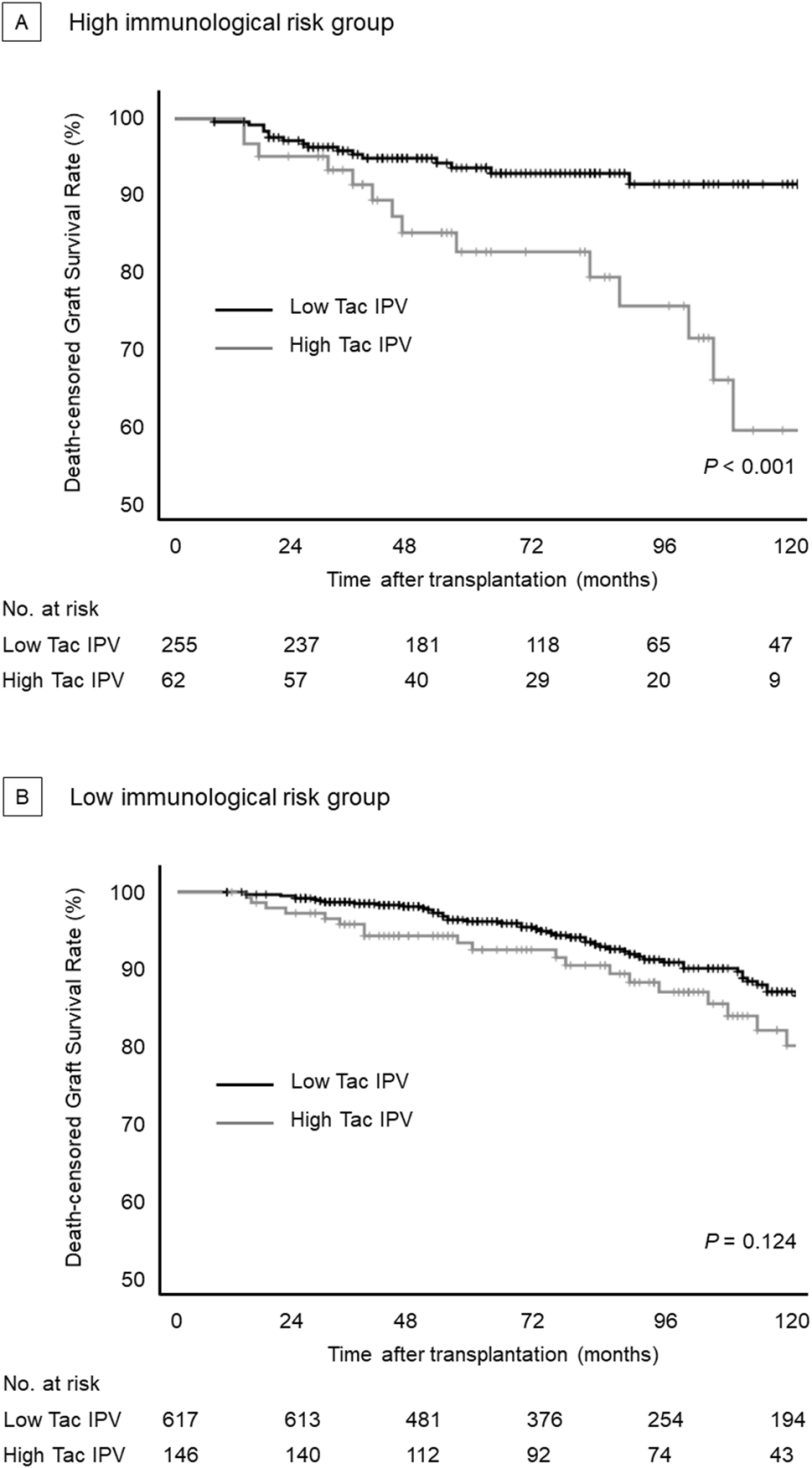
Overall graft survival according to tacrolimus IPV (**A**) high immunological risk group, (**B**) low immunological risk group.

Throughout the follow-up period, 130 graft losses occurred (93 graft failures and 37 patient deaths). The association between tacrolimus IPV and death-censored graft survival was evident in the high immunological risk group (Fig. 1). Death-censored graft survival in the high immunological risk group was significantly impaired with high tacrolimus IPV (*P* < 0.001). A multivariable Cox regression analysis confirmed that high tacrolimus IPV was independently associated with graft failure in the high risk group [hazard ratio (HR), 2.90; 95% confidence interval (CI), 1.42–5.95; *P* = 0.004; Table 3]. Tacrolimus IPV was also associated with an increased risk of graft failure when assessed as continuous variables. In contrast, death-censored graft survival of the low immunological risk group was not significantly different according to tacrolimus IPV (*P* = 0.066). In the low-risk group, elderly recipient, estimated glomerular filtration rate (eGFR) at 1-year post-KT, deceased donor, and late-onset graft rejection were significant risk factors for graft failure, whereas high tacrolimus IPV was not associated with graft failure (Table 4).

**Table 3:** Risk factors for graft loss in the high immunological risk group.

Variables	Univariate		Multivariate	
	HR (95% CI)	*P*	HR (95% CI)	*P*
Elderly recipient (Age ≥ 60 years)	3.631 (1.699–7.761)	0.001	3.095 (1.267–7.558)	0.013
eGFR at 1 year, mL/min/1.73m^2^	0.964 (0.946–0.982)	< 0.001	0.966 (0.947–0.986)	0.001
Re-transplant	1.257 (0.588–2.685)	0.556		
Donor age, years	1.043 (1.011–1.076)	0.007	0.990 (0.957–1.025)	0.581
Deceased donor	2.399 (1.153–4.993)	0.019	1.892 (0.849–4.213)	0.119
Mean TAC trough concentration	1.033 (0.867–1.231)	0.712		
High TAC IPV (CV > 30%)	3.172 (1.578–6.382)	0.001	2.904 (1.417–5.950)	0.004
Late-onset graft rejection	3.827 (1.843–7.947)	< 0.001	3.237 (1.511–6.932)	0.003

*eGFR* Estimated glomerular filtration rate, *TAC* Tacrolimus, *IPV* Intrapatient variability, *CV* Coefficient of variation.

**Table 4:** Risk factors for graft loss in the low immunological risk group.

Variables	Univariate		Multivariate	
	HR (95% CI)	*P*	HR (95% CI)	*P*
Elderly recipient (Age ≥ 60 years)	2.033 (1.120–3.690)	0.020	2.259 (1.210–4.219)	0.011
eGFR at 1 year, mL/min/1.73m^2^	0.956 (0.943–0.969)	< 0.001	0.970 (0.956–0.984)	< 0.001
Re-transplant	0.668 (0.244–1.830)	0.433		
Donor age, years	1.032 (1.012–1.053)	0.002	1.010 (0.987–1.034)	0.397
Deceased donor	1.741 (1.083–2.799)	0.022	1.717 (1.024–2.882)	0.041
Mean TAC trough concentration	1.019 (0.918–1.132)	0.721		
High TAC IPV (CV > 30%)	1.565 (0.967–2.535)	0.068	1.040 (0.629–1.720)	0.879
Late-onset graft rejection	7.219 (4.653–11.201)	< 0.001	6.960 (4.356–11.120)	< 0.001

*eGFR* Estimated glomerular filtration rate, *TAC* Tacrolimus, *IPV* Intrapatient variability, *CV* Coefficient of variation.

### Tacrolimus IPV and graft rejection

**Figure 2:**
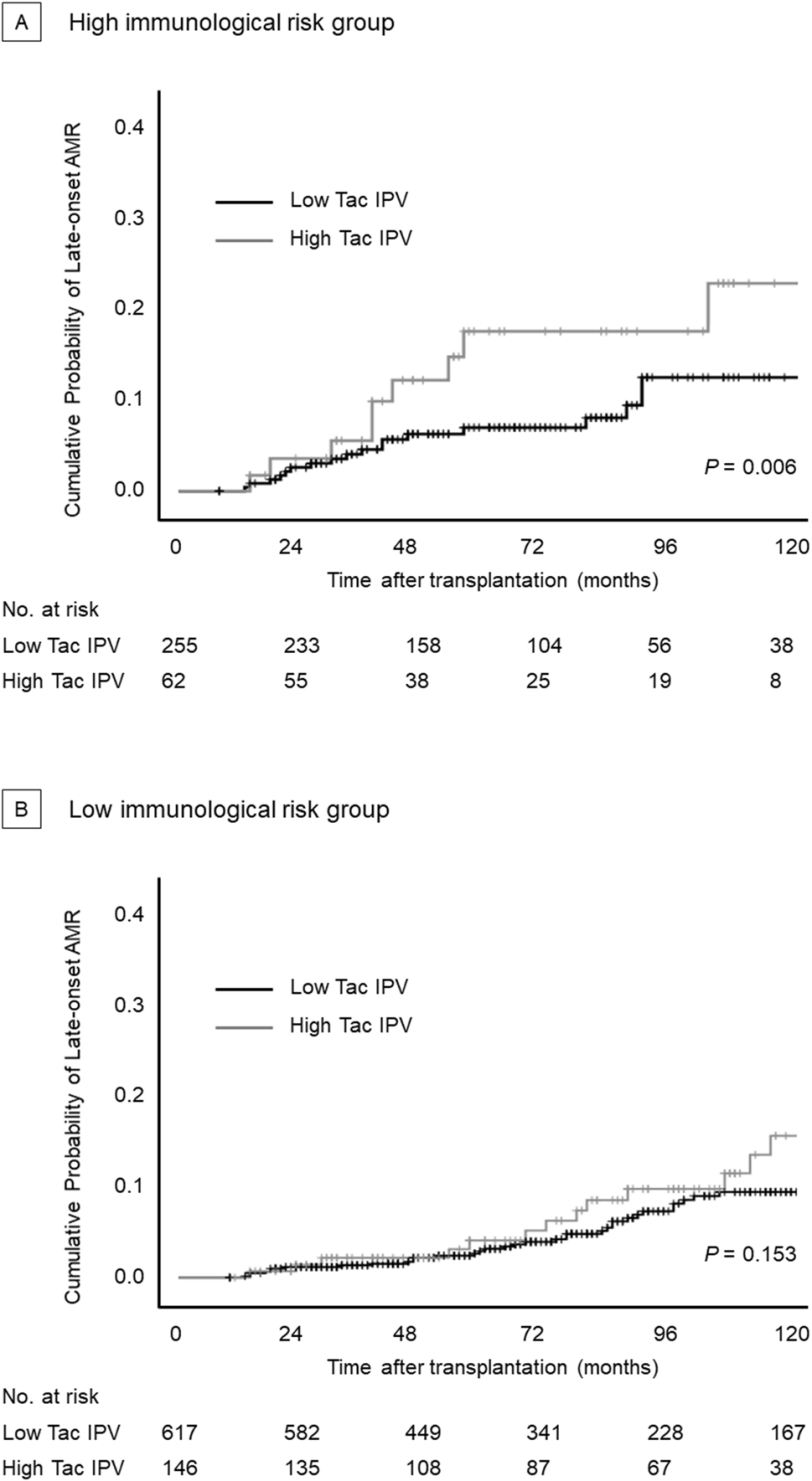
Cumulative probability of late-onset antibody-mediated rejection according to tacrolimus IPV (**A**) high immunological risk group, (**B**) low immunological risk group.

During the follow-up period, 294 graft rejection episodes [171 antibody-mediated rejection (AMR) and 123 T-cell mediated rejection (TCMR)] occurred in 200 recipients. A total of 163 late-onset rejection (> 12 months after transplant, 51 active AMR, 58 chronic active AMR, 4 chronic inactive AMR, and 50 TCMR) episodes occurred in 115 patients. Overall cumulative probabilities for late-onset AMR in high immunological risk group were significantly higher than in low immunological risk group (*P* = 0.035), whereas cumulative probabilities for late-onset TCMR between two groups were not significantly different (*P* = 0.533). The association between tacrolimus IPV and late-onset AMR was significantly different between the two groups (Fig. 2). High tacrolimus IPV was significantly associated with increased risk of late-onset AMR in the high-risk group (*P* = 0.006). In the low-risk group, high tacrolimus IPV was not associated with late-onset AMR (*P* = 0.153). High tacrolimus IPV was not associated with late-onset TCMR in both groups.

## Discussion

To date, there has been limited novel immunosuppressive drug development to improve long-term transplant outcomes^6^. Until new therapeutic drugs are available, optimization of current immunosuppression based on immunological risk profile remains the only option to improve long-term graft outcomes after KT^17^. In the present study, high tacrolimus IPV was significantly associated with an increased risk of graft failure in the high immunological risk group, whereas in the low immunological risk group it was not associated with graft failure. In addition, significant association between high tacrolimus IPV and late-onset AMR was observed only in the high-risk group.

Tacrolimus is the most commonly prescribed immunosuppressive drug for KT patients^1^. It is known that tacrolimus is safe and effective only in a narrow therapeutic window. However, optimal tacrolimus trough levels have not been clearly defined. In addition to a narrow and poorly defined therapeutic window, clinical use of tacrolimus is complicated by significant IPV^11^. Since Borra et al. first described the negative effect of high tacrolimus IPV on graft outcomes, there is a growing body of literature to support the association between high tacrolimus IPV and deleterious graft outcomes^13,14,18^. Previous studied have suggested that high tacrolimus IPV leads to inferior graft outcomes due to alloimmune-mediated injury^12,19^. Therefore, the effects of tacrolimus IPV on graft outcome might be more pronounced in patients with high immunological risk than in those with low immunological risk. To utilize tacrolimus IPV measurements for tailored immunosuppression treatment, the patient’s immunological risk should be taken into account.

Several studies suggested that tacrolimus underexposure significantly increased the risk of alloimmune-mediated injury according to immunological risk^20^. Wiebe et al. reported a significant impact of tacrolimus trough concentrations on the development of de novo DSA based on immunological risk as determined by HLA-DR/DQ epitope mismatch^15^. However, none of these studies analyzed tacrolimus IPV, which is associated with inferior graft outcomes. Our findings focused on the tacrolimus IPV extend previous findings of the importance of immunological risks in tailored immunosuppressive strategies. In the present study, clinical association between tacrolimus IPV and graft outcomes was more evident in patients with high immunological risk than in those with low immunological risk. Multivariable analysis revealed that high tacrolimus IPV was significantly associated with graft loss in the high immunological risk group. In the high-risk group, patients with high tacrolimus IPV were also at greater risk of AMR. Patients with low immunological risk were more likely to tolerate high tacrolimus IPV without developing AMR. This study highlights the importance of assessing immunological risk when creating tailored immunosuppressive strategies. To the best of our knowledge, this is the first study to assess the effect of tacrolimus IPV across a range of immunological risk groups.

In the present study, immunological risk was stratified by pre-transplant PRA and DSA, which are the most commonly used tests for determining immunological risk in clinical practice. We acknowledge that these tests for identifying patient risk for memory or alloimmune response is limited and the cut-off is arbitrary^21^. We attempted to minimize misclassification by excluding immunologically incompatible KTs and zero-HLA mismatch KTs. Although significant advances in immunological tests, including complement fixation assays, non-HLA antibodies, and HLA molecular mismatch have permitted more comprehensive immunological risk assessment, none of the tests are widely used in current clinical practice^15,22–24^. Evidence supporting the added clinical benefit of these tests over traditional immunological tests remains controversial. Further studies are needed to evaluate the effects of tacrolimus IPV based on more accurate immunological assessment.

Several possible mechanisms for high tacrolimus IPV include medication non-adherence, drug-drug interaction, food intake, gastrointestinal disorders, and changing hematocrit^18,25^. Although medication non-adherence is a major determinant of high IPV, some degree of IPV has been identified, even in highly adherent patients^26^. In the present study, low hematocrit level at the first post-transplant year and high tacrolimus concentration to dose ratio were significantly associated with high tacrolimus IPV. This finding is consistent with the fact that tacrolimus mainly distributes into and binds to erythrocytes. High tacrolimus IPV, irrespective of its cause, is an important risk factor for poor graft outcomes. Previous studies have shown that adherence-enhancing interventions can improve tacrolimus IPV^18,27^. Taken together, this suggests that patients classified as high immunological risk need tight monitoring and interventions such as educational support and simplified drug regimens.

The present study has some limitations that warrant consideration. First, retrospective single-center investigation limits the generalizability of this study. However, this made it possible to maintain homogeneity in immunosuppressive regimen, patient education, and follow-up protocol. Second, information about adherence is lacking. Although medication non-adherence is a major determinant of high IPV, it is not the sole cause of high IPV^26^. In addition, objective adherence is difficult to measure in routine clinical practice. We attempted to evaluate other potential risk factors for high tacrolimus IPV. Third, de novo DSA were not systematically determined. We adopted de novo DSA monitoring in 2011, but prior to this time such measurements were limited due to national health insurance coverage and reimbursement. Despite the clinical significance of de novo DSA, there remains no consensus on how best to monitor de novo DSA after transplantation^21^. Further prospective research is needed to identify the clinical significance of tacrolimus IPV on developing de novo DSA according to immunological risk.

In conclusion, high tacrolimus IPV significantly increases the risk of graft loss and late-onset AMR after KT, especially in high immunological risk patients. Our results support evaluation of tacrolimus IPV in the context of each patient’s immunological risk. Using tacrolimus IPV to individualize immunosuppressive treatment and monitoring may improve long-term graft outcomes. Future clinical trials are warranted to fully assess this approach.

## Methods

### Study population

**Figure 3:**
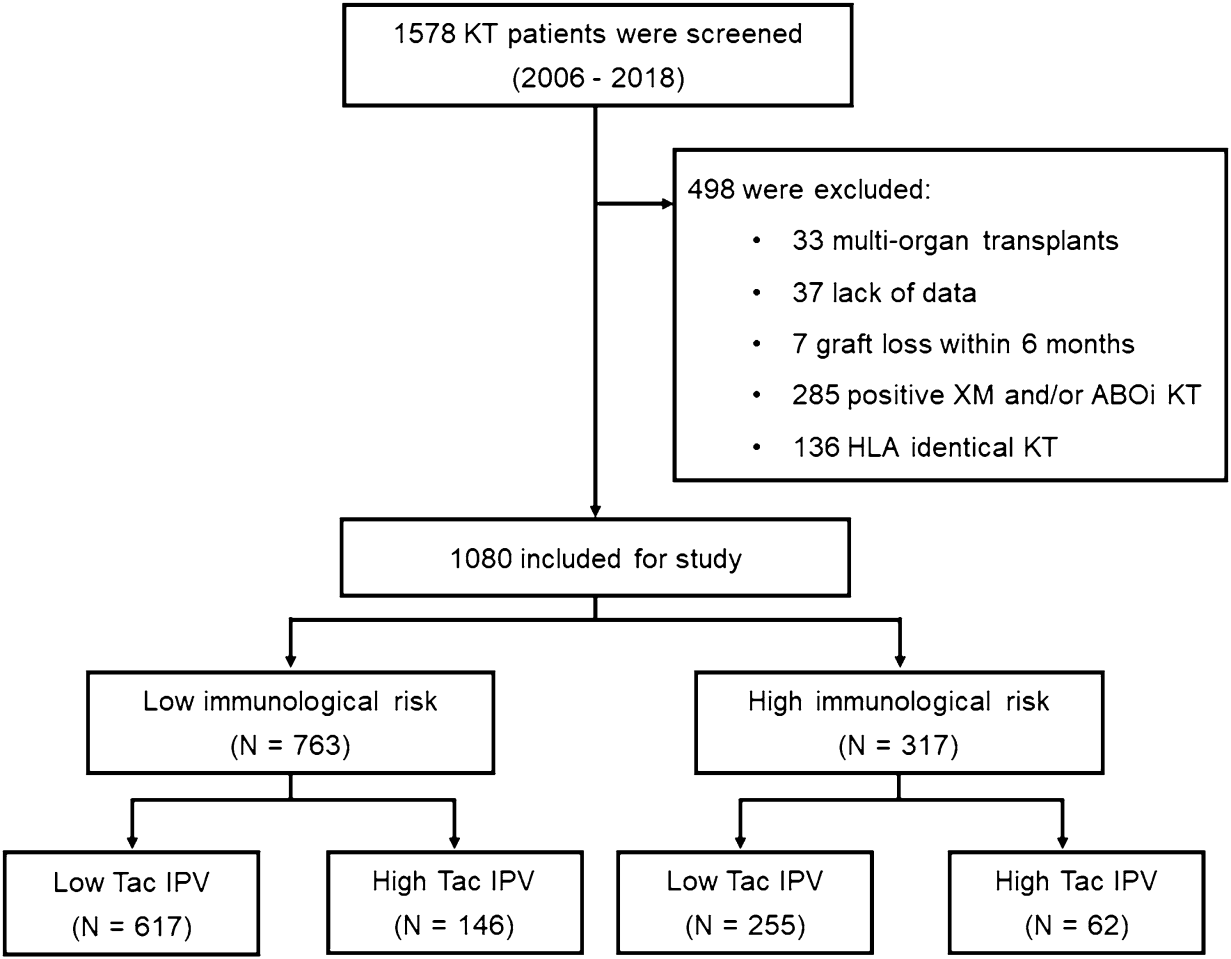
Study diagram.

A total of 1578 patients (aged ≥ 18 years) who underwent KT under tacrolimus-based immunosuppression between January 2006 and December 2018 at the Severance Hospital, Seoul, Republic of Korea, were initially screened. Patients who underwent multi-organ transplantation, patients with positive crossmatch and/or ABO-incompatible KT, and zero-HLA mismatch KT patients were excluded. Patients who experienced graft loss within 6 months or who lacked sufficient data were also excluded. After applying these restrictions, 1080 patients were ultimately included in the study. These patients were grouped into low- and high immunological risk groups based on pre-transplant peak PRA and presence of DSA. High immunological risk was defined as PRA ≥ 20% or the presence of DSA (Fig. 3).

### Immunosuppression

Immunosuppression was performed according to center protocol^28^. Most patients received induction immunosuppression with basiliximab (20 mg on days 0 and 4 post-transplant) or anti-thymocyte globulin (1.5 mg/kg per day for 4 days). Maintenance immunosuppression for all patients consisted of tacrolimus, prednisolone, and mycophenolate mofetil (MMF). The initial tacrolimus dosage (0.1 mg/kg) was administered orally twice daily. Subsequent doses were adjusted to maintain a target trough concentration between 5 and 8 ng/ml. The initial dose of methylprednisolone (500–1000 mg) was gradually reduced and replaced with oral prednisolone (5–10 mg/day) during the first 3 weeks after transplantation. MMF was started at 1.0–1.5 g/day and subsequently adjusted to minimize adverse events such as neutropenia or gastrointestinal side effects.

### Clinical and laboratory measurements

Pre-transplant PRA and DSA were measured as described previously^24^. Briefly, PRA testing was performed using Luminex PRA assay kits (LIFECODES LifeScreen Deluxe and Class I and Class II ID, Immucor Transplant Diagnostics, Stamford, CT, USA) and was presented as percent PRA. DSA were detected using Lifecodes LSA Class I and Class II kits (Immucor Transplant Diagnostic) or LabScreen Single Antigen (One Lambda, Canoga Park, CA, USA) according to the manufacturers’ protocols. The strength of each DSA was determined at the maximum mean fluorescence intensity (MFI) value and a MFI of > 1000 was considered positive.

Routine biochemical tests, including the assessment of tacrolimus trough concentrations, were performed every month during the first year post transplantation and every 3 months thereafter. Tacrolimus trough concentrations were determined using a microparticle enzyme immunoassay [Tacrolimus II MEIA/IMx analyzer (Abbott Laboratories, Chicago, IL, USA) until May 8, 2008; Dimension RxL (Siemens, Munich, Germany) between May 9, 2008 and February 25, 2013; Architect i2000 (Abbott Laboratories) from February 26, 2013 onward]. Erroneously high tacrolimus concentrations (> 20 ng/mL) resulting from taking morning doses before blood sampling were excluded. Tacrolimus IPV was estimated by calculating the CV according to the following equation: CV (%) = (standard deviation/mean tacrolimus trough concentration) × 100. Mean concentrations were calculated using outpatient tacrolimus concentrations between 6 and 12 months^14^. We used a CV cutoff value of 30% for high tacrolimus IPV^12,26^.

eGFR was evaluated using the Chronic Kidney Disease Epidemiology Collaboration equation^29^. Renal biopsies were performed in cases of acute allograft dysfunction (> 30% increase in serum creatinine levels compared with baseline or proteinuria > 500 mg/day). Allograft biopsy samples were processed using light, immunofluorescent, and electron microscopy at the time of biopsy. All allograft rejections were confirmed by biopsy and classified into AMR and TCMR according to the most recent Banff criteria at the time of biopsy^30^.

### Definition and study endpoints

The primary study endpoint was death-censored graft survival. The secondary endpoints included late-onset AMR and TCMR. Graft failure was defined as the return to long-term dialysis or re-transplantation. Graft survival was calculated from the date of transplantation to the date of graft failure, loss to follow-up, or December 31, 2020 (the end of the follow-up period). In cases of death with a functioning graft, graft survival was censored at the time of death. Late-onset rejection was defined as any biopsy-confirmed rejection that occurred more than 12 months post-transplantation.

### Statistical analysis

Data were expressed as frequency, mean, and standard deviation, or as the median and IQR, depending on the data type. Chi-square or Fisher’s exact tests were used as appropriate to compare categorical variables. Continuous variables were compared using Student’s t-test for parametric data or the Mann–Whitney test for nonparametric data. Multivariable logistic regression was performed using the high tacrolimus IPV (CV > 30%) as an outcome variable. Covariates included baseline characteristics and laboratory findings at 12 months post-transplantation. Death-censored graft survival and cumulative probability of AMR were analyzed using Kaplan–Meier curves and the log-rank test. Cox proportional hazard regression models were used to evaluate the associations between tacrolimus IPV and time to graft loss. Statistical analyses were performed using SPSS software (version 25.0; SPSS Inc., Chicago, IL, USA). *P* < 0.05 was considered significant.

### Ethics statement

All study procedures were conducted in accordance with the Declaration of Helsinki and were approved by the Institutional Review Board of Severance Hospital (2020-2851-001). Informed consent was waived by the Institutional Review Board of Severance Hospital because of the study’s retrospective design.

## Data Availability

The datasets generated during and/or analyzed during the current study are available from the corresponding author on reasonable request.

## References

[1] Hart A (2020). OPTN/SRTR 2018 annual data report: kidney. Am. J. Transpl..

[2] Lodhi SA, Lamb KE, Meier-Kriesche HU (2011). Solid organ allograft survival improvement in the United States: the long-term does not mirror the dramatic short-term success. Am. J. Transpl..

[3] El-Zoghby ZM (2009). Identifying specific causes of kidney allograft loss. Am. J. Transpl..

[4] Sellarés J (2012). Understanding the causes of kidney transplant failure: the dominant role of antibody-mediated rejection and nonadherence. Am. J. Transpl..

[5] Wekerle T, Segev D, Lechler R, Oberbauer R (2017). Strategies for long-term preservation of kidney graft function. Lancet.

[6] Stegall MD, Morris RE, Alloway RR, Mannon RB (2016). Developing new immunosuppression for the next generation of transplant recipients: the path forward. Am. J. Transpl..

[7] Lim MA, Kohli J, Bloom RD (2017). Immunosuppression for kidney transplantation: Where are we now and where are we going?. Transpl. Rev. (Orlando).

[8] O’Connell PJ (2017). Clinical trials for immunosuppression in transplantation: the case for reform and change in direction. Transplantation.

[9] Axelrod DA (2016). National variation in use of immunosuppression for kidney transplantation: a call for evidence-based regimen selection. Am. J. Transpl..

[10] Ekberg H (2007). Reduced exposure to calcineurin inhibitors in renal transplantation. N. Engl. J. Med..

[11] Brunet M (2019). Therapeutic drug monitoring of tacrolimus-personalized therapy: second consensus report. Ther. Drug. Monit..

[12] Rodrigo E (2016). Within-patient variability in tacrolimus blood levels predicts kidney graft loss and donor-specific antibody development. Transplantation.

[13] Borra LC (2010). High within-patient variability in the clearance of tacrolimus is a risk factor for poor long-term outcome after kidney transplantation. Nephrol. Dial. Transpl..

[14] Gonzales HM (2020). A comprehensive review of the impact of tacrolimus intrapatient variability on clinical outcomes in kidney transplantation. Am. J. Transpl..

[15] Wiebe C (2017). Class II eplet mismatch modulates tacrolimus trough levels required to prevent donor-specific antibody development. J. Am. Soc. Nephrol..

[16] Pratschke J (2016). Immunological risk assessment: the key to individualized immunosuppression after kidney transplantation. Transpl. Rev. (Orlando).

[17] Abramowicz D (2018). Recent advances in kidney transplantation: a viewpoint from the Descartes advisory board. Nephrol. Dial. Transpl..

[18] Kuypers DRJ (2020). Intrapatient variability of tacrolimus exposure in solid organ transplantation: a novel marker for clinical outcome. Clin. Pharmacol. Ther..

[19] Davis S (2018). Lower tacrolimus exposure and time in therapeutic range increase the risk of de novo donor-specific antibodies in the first year of kidney transplantation. Am. J. Transpl..

[20] Richards KR (2014). Tacrolimus trough level at discharge predicts acute rejection in moderately sensitized renal transplant recipients. Transplantation.

[21] Tambur AR (2018). Sensitization in transplantation: assessment of risk (STAR) 2017 working group meeting report. Am. J. Transpl..

[22] Loupy A (2013). Complement-binding anti-HLA antibodies and kidney-allograft survival. N. Engl. J. Med..

[23] Dragun D (2005). Angiotensin II type 1-receptor activating antibodies in renal-allograft rejection. N. Engl. J. Med..

[24] Lee J (2017). The clinicopathological relevance of pretransplant anti-angiotensin II type 1 receptor antibodies in renal transplantation. Nephrol. Dial. Transpl..

[25] Størset E (2014). Importance of hematocrit for a tacrolimus target concentration strategy. Eur. J. Clin. Pharmacol..

[26] Leino AD (2019). Assessment of tacrolimus intrapatient variability in stable adherent transplant recipients: Establishing baseline values. Am. J. Transpl..

[27] Kuypers DR (2013). Improved adherence to tacrolimus once-daily formulation in renal recipients: a randomized controlled trial using electronic monitoring. Transplantation.

[28] Lee J (2017). Rituximab and hepatitis B reactivation in HBsAg-negative/ anti-HBc-positive kidney transplant recipients. Nephrol. Dial. Transpl..

[29] Levey AS (2009). A new equation to estimate glomerular filtration rate. Ann. Intern. Med..

[30] Haas M (2018). The Banff 2017 Kidney Meeting Report: Revised diagnostic criteria for chronic active T cell-mediated rejection, antibody-mediated rejection, and prospects for integrative endpoints for next-generation clinical trials. Am. J. Transpl..

